# Comparison of physical electrical conductivity and acupuncture *de-qi* sensation between stainless steel needling and supercritical fluid-treated needling

**DOI:** 10.1016/j.bj.2020.11.010

**Published:** 2020-11-21

**Authors:** Ting-Hao Wang, Ming-Hui Wang, Chih-Cheng Shih, Ying-Hsin Lu, Hao-Xuan Zheng, Yi-Ting Tseng, Wen-Long Hu, Ting-Chang Chang, Yu-Chiang Hung

**Affiliations:** a Department of Chinese Medicine, Chang Gung Memorial Hospital at Kaohsiung, Kaohsiung, Taiwan; b Department of Materials and Optoelectronic Science, National Sun Yat-Sen University, Kaohsiung, Taiwan; c Department of Physics, National Sun Yat-Sen University, Kaohsiung, Taiwan; d Fooyin University College of Nursing, Kaohsiung, Taiwan; e Kaohsiung Medical University of Medicine, Kaohsiung, Taiwan; f School of Chinese Medicine, College of Medicine, Chang Gung University, Taoyuan, Taiwan

**Keywords:** Acupuncture needle, Supercritical fluid, *De-qi* sensation, Chinese medicine

## Abstract

**Background:**

While acupuncture has been used for thousands of years, modern technology to develop new needle materials has rarely been discussed. We aim to explore a new acupuncture needle material and compare the differences in the needling sensations between the acupuncture needle surface treated with nitrogen applied supercritical fluid (SCF–N) and conventional stainless steel needles.

**Methods:**

This was a double-blind cohort study. The acupuncture needles were randomly used in this experiment, including the SCF-N-treated needles and the control stainless steel needles. LI 4 (Hegu) and LI 11 (Quchi) acupuncture points in the Yangming Large Intestine Meridian of Hand were treated. Physical electrical resistance, scanning electron microscopy, energy dispersive spectrometry, and visual analog scale (VAS) score including the sensations of soreness, numbness, distention, and heaviness were assessed.

**Results:**

The proportion of nitrogen (N) was significantly higher in the SCF-N-treated needles than in the stainless steel needles group (2.3 ± 0.2% vs 0.0 ± 0.0%, *P* < 0.01). The cumulative *de-qi* sensation score at the LI 4 Hegu acupoint (1.87 ± 1.88 vs 1.54 ± 1.62, *P* = 0.014), especially the sensation of soreness score (2.76 ± 2.06 vs 2.13 ± 1.85, *P* = 0.045), revealed statistically significant differences between both groups. SCF-N surface treatment of acupuncture needles may lower the electrical resistance more than the control stainless steel needles (24.67 ± 0.88 kW vs 26.45 ± 0.75 kW, *p* < 0.01).

**Conclusion:**

Acupuncture needles modified with SCF-N surface treatment can enhance *de-qi* sensations to improve electrical conductivity of the meridian and therapeutic effects on the Yangming Large Intestine Meridian of Hand. SCF-N surface treated needles can be as a new acupuncture needle material in the future.

**At a glance of commentary**

**Scientific background on the subject**

Through the good fluidity and solubility of supercritical fluid, acupuncture needles were modified with supercritical fluid-N treated in this study. Acupuncture needle modified with SCF-N surface treatment can enhance de-qi sensations, especially the sensation of soreness, and improve electrical conductivity of the meridian.

**What this study adds to the field**

Acupuncture needles changes from stone, ceramic, bamboo, bronze, iron, gold, and silver, to the stainless steel currently in use. The SCF technology with gas-like diffusivity and liquid-like density in the supercritical phase can be used for acupuncture needle modifications. SCF-N-treated needles can be as a new acupuncture needle material.

Acupuncture therapy involves inserting thin needles into acupoints, which has been used in Chinese communities for more than three thousand years. Before the 1970s, it was globally known as an instrument for anesthesia [1]. Acupuncture is not only known as an important therapy for musculo-skeletal conditions, but is also widely used to treat internal diseases, such as immune system dysfunctions and gastrointestinal diseases, as well as ailments in gynecological and neurological functioning [[2], [3], [4], [5]]. Acupuncture is a kind of specialized medicine that is easy to perform, widely used, fast, safe and effective, and is well received by the general public. Though practitioner skills are paramount in producing the therapeutic effect, the importance of appropriate needles should not be underestimated. Throughout the history of the evolution of acupuncture needles, we can see material changes from stone, ceramic, bamboo, bronze, iron, gold, and silver [6,7], to the stainless steel currently in use. Some research has examined the surface conditions and other various physical properties of sterilized single-use stainless steel acupuncture needles and highlighted the need for improved quality control of acupuncture needles [6]. Newer materials and techniques are also constantly developing [8].

Supercritical fluid (SCF) technology has recently attracted attention in several research fields, including pharmaceutics. SCFs utilize solvent properties above their critical temperature and pressure to exhibit liquid- and gas-like properties [9]. SCF can flow out of solids like a gas and dissolve substances like a liquid. Over the past 35 years, SCF has been applied in analytical chemistry for various processes such as chromatographic extraction of drugs, surface modification of medical material, plasticization of polymers, nanosizing and nanocrystal modification [10]. This technology has also been applied to environmental chemistry, food and polymer chemistry, and pharmaceutical and agricultural research [11]. However, to our knowledge, there have been no studies applying this technology to improve acupuncture needles.

There are few elements used in the SCF technique. The needles in this experiment is sterilized and disposable. As acupuncture is an invasive technique which involves insertion into the human body with needles contacting the body fluid circulation system, the safety of the needle becomes important to avoid any adverse reactions in the human body. Hence, the element used in this experiment for surface treatment of the needles was nitrogen (N), which is the most common element in the world. As mentioned in an earlier study, low resistance and high capacitance are generally accepted as electrical characteristics of meridians and acupoints [12]. Research also expected that this higher electrical conductivity and low resistance would improve the effectiveness of electro-acupuncture [8]. Electro-acupuncture is widely used to strengthen the effect of acupuncture. In this study, electrical conductivity and resistance of the needles were used as standards for assessing therapeutic effects [13,14]. The conductivity and resistance of SCF-N-treated needles were compared with those of untreated needles. The aim of this experiment was to test the differences in physical composition and properties between SCF-N-treated acupuncture needles and control stainless steel needles in order to test the possibility of SCF treatment as a novel means of improving the effect of acupuncture therapy.

## Material and methods

### Participant selection

This clinical trial was approved by the Institutional Review Board of Chang Gung Medical Foundation (IRB permit no. 201700722A3) and registered at ClinicalTrials.gov (identification number NCT04073277). Written informed consent was obtained from all participants before enrollment. The study was done in accordance with the Revised Standards for Reporting Interventions in Clinical Trials of Acupuncture (STRICTA) [15], extending the Consolidated Standards of Reporting Trials (CONSORT) statement [16].

### Inclusion and exclusion criteria

According to a prospective survey, despite its benefits, acupuncture could also lead to some side effects [17]. It may sometimes induce either local or systemic adverse reactions as an invasive treatment [[17], [18], [19]]. Moreover, a systemic review shows that life-threatening events may also develop, albeit rarely [19]. Bleeding and hematoma are the most common adverse reactions. Volunteers with bleeding tendencies (platelet counts less than 20000 and/or thrombocytopenic purpura) were excluded. Volunteers with chronic medical conditions who were prescribed anti-coagulants were also excluded. In addition, pregnant women and volunteers with pacemakers were excluded.

### Experimental design

This was a double-blind prospective cohort study. The acupuncture needles were first separated into two groups. One group of needles was subjected to SCF-N treatment while the other was not. The needles were then randomly analyzed with scanning electron microscope and energy dispersive X-ray spectroscopy to ensure quality and minimize experimental error. For each participant, one hand was randomly assigned to the treatment group with SCF-N-treated acupuncture needles and the other hand was assigned to the control group with the stainless steel needles. The time interval between the acupuncture treatment of the two groups was about 2 h. In order to examine and distinguish the difference between the two groups, *de-qi* sensation VAS score was self-reported and recorded by the volunteers during needle insertion and the electrical resistance was measured by electrical measurement.

### Acupuncture needles

The acupuncture needles used in this experiment, including the SCF-N-treated needles and the control stainless steel needles, were produced under the same conditions, in the same factory (Dong Bang Acupuncture Inc.), and on the same day, to minimize experimental error.

**Fig. 1:**
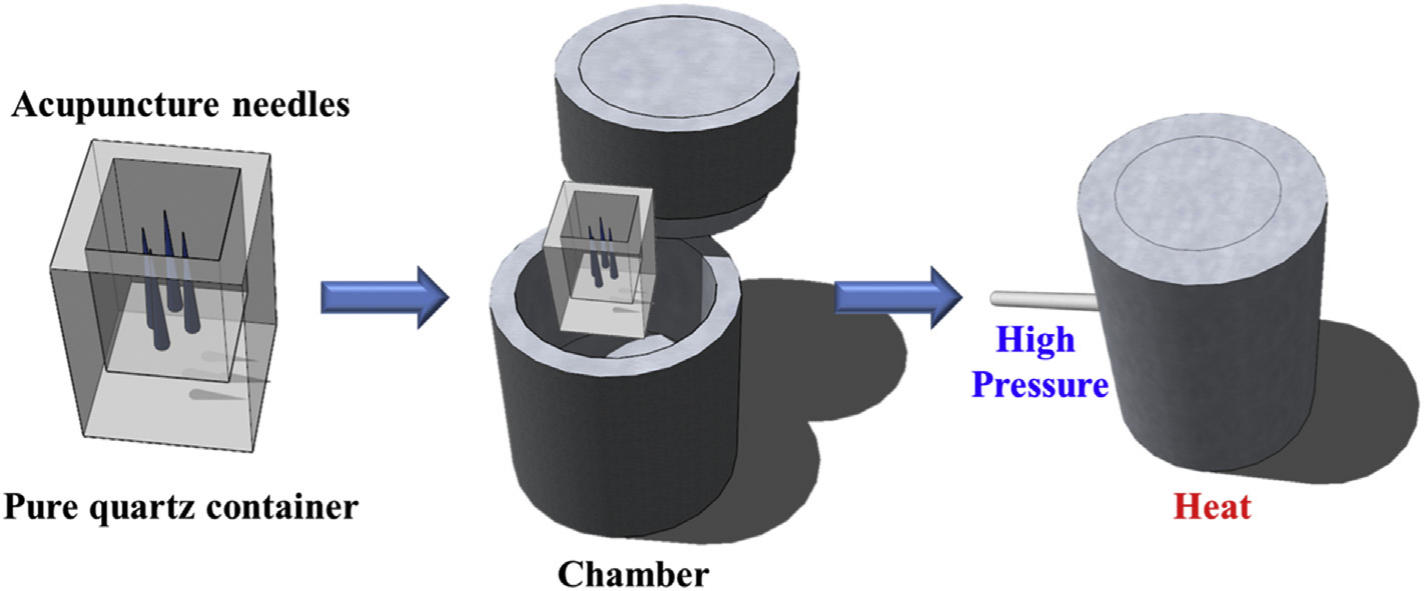
Supercritical fluid equipment used in this experiment, with supercritical reaction progressed by a high-pressure pump and the higher temperature of the chamber.

The process of SCF treatment is shown in Fig. 1. First, the stainless steel chamber and quartz carrier were sterilized using alcohol and autoclaving. Next, the needles were placed on the quartz carrier with the tip pointing upwards and then placed in the chamber and covered. Carbon dioxide was used to remove atmospheric components from the chamber and the required volume of ammonia gas was introduced. The pressure was increased up to 3000 pound per square inch (psi) and temperature up to 120 °C. An hour later, the pressure was relieved and the process of SCF treatment was complete. After completion of the treatment, the needles were then randomly selected for material analysis and electrical measurement. Following this, the needles were stored in a vacuum bag. This was done to reduce the influence of atmospheric contaminants on the needles after treatment which might result in oxidation and rusting. Finally, there was no heterogeneous contact between the inorganic metal and the acupuncture needle.

### Measurement equipment

**Fig. 2:**
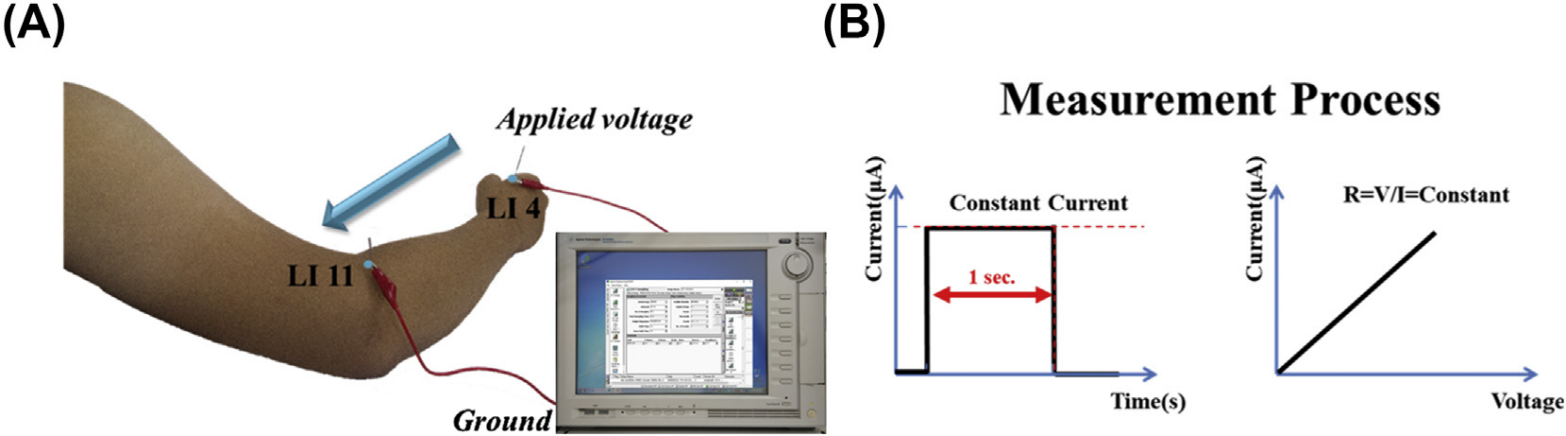
(A) LI4 (Hegu) and LI11 (Quchi) as the acupuncture points. (B) The current, voltage, and electrical resistance measurements.

Fig. 2 describes the setting of this experiment. Fig. 2(A) shows the two chosen LI4 (Hegu) and LI11 (Quchi) acupoints. LI4 and LI11 are acupuncture points in the Yangming Large Intestine Meridian of Hand. LI4 is on the dorsum of the hand, midway between the 1st and 2nd metacarpal bones, approximately in the middle of the 2nd metacarpal bone on the radial side. When the elbow is flexed, LI11 is the midpoint between the lateral end of the transverse cubical crease and the lateral epicondyle of the humerus. These two acupoints were chosen for ease and convenience of precise localization. In this experiment, each volunteer underwent acupuncture on one side of the arm with the stainless steel needles and then on the other side with the SCF-N-treated needles. The needles were sequentially inserted at 90° into the chosen points using the promotion needling technique in which the needles were twisted, slightly lifted, shaken, or re-inserted for 30 s without strong intense stimulation. First, the inserted needles were attached to the measuring equipment. The equipment used in this experiment was an Agilent B1500 semi-conductor analyzer and Cascade M150 microprobe station, a system to measure electrical resistance with high accuracy (Keysight Technologies, Inc.). Second, the current was set up to measure the variation in the electrical resistance. Upon completion of the treatment, subject self-reported and recorded the modified VAS score based on the multiple *de-qi* sensations experienced. To avoid bias, the experiment was double-blind, meaning that except for the experiment designer, the doctor and volunteers were both unaware of the needle material. Fig. 2(B) shows the current output and electrical resistance, which also represented the resistance of the meridian. Electrical resistance was measured by applying a fixed electric current (110 μA) for 1 s and recording the I–V curve. A linear relationship of voltage = current∗resistance was expected.

### Scanning electron microscope and energy dispersive spectrometer

A JEOL JSM 6330 F Field Emission Scanning Electron Microscope (SEM) at 30 kV with energy dispersive X-ray analysis system (EDS) was used to confirm the surface properties of the material. The electron signal during SEM scanning can be used to observe whether or not the needle tip surface before and after SCF treatment is different. Based on the results of the EDS, the composition of the needle can be distinguished, and are presented below.

### Modified visual analog scale (VAS) score

To evaluate the effect of acupuncture therapy, we often assess the needling sensation as a valuable factor. The *de-qi* sensation, originating from Neijing, is regarded as the needle sensation indicative of successful acupuncture which can elicit intrinsic changes in the human body [[20], [21], [22]]. VAS is a self-reporting scale used in acupuncture to assess patient's physical sensations, and is one of the most widely used scales [20,21]. This study showed that the most common types of needle sensation reported by the 20 volunteers were distension, soreness, heaviness, or numbness [20,21]. Some review articles have shown that there are many scales and questionnaires, including both qualitative and quantitative evaluation of the *de-qi* sensation [23,24]. In this study, we combined the VAS score and assessment of the four *de-qi* sensations to evaluate participants' experience, which can also represent the therapeutic effects.

### Statistical analysis

All data evaluations in this study were repeated three times, and are presented as mean ± standard deviation. Analysis of variance and paired *t* test were used in this study. Differences were considered to be statistically significant at a *P* value of <0.05. All analyses were performed with SPSS for Windows, version 17.0 (Statistics 17.0, SPSS, IBM, New York, NY).

## Results

After certain initial assessments, 20 volunteers who met the inclusion criteria were recruited. The 15 men and 5 women volunteers with ages ranging from 20 to 30 years (24.7 ± 2.6 years) were all well-informed about this project and signed the consent before the experiment.

### Scanning electron microscope and energy dispersive spectrometer

**Fig. 3:**
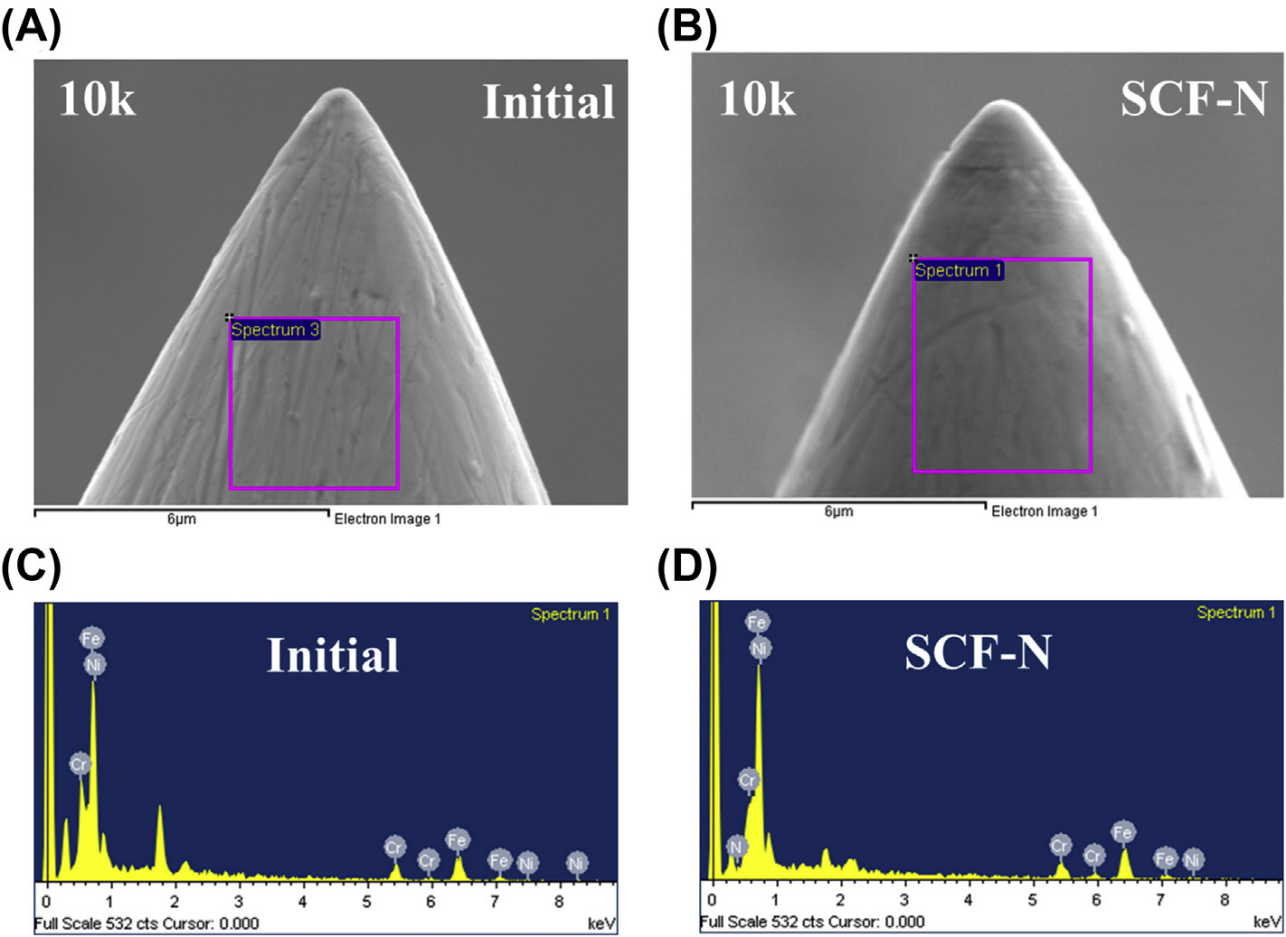
Scanning electron microscope images of (A) untreated and (B) treated needles. The tip appearance of SCF-N-treated needles reveals no significant malformation. Energy dispersive spectrometer signal results for (C) untreated and (D) treated needles.

Using a scanning electron microscope (SEM), the different surface features between the two groups of needles were revealed at a magnification of 10,000 × , as shown in Fig. 3(A) and (B). The tip appearance of the SCF-N-treated needles revealed no significant malformation. In order to analyze the composition of the substances, an energy dispersive spectrometer (EDS) was used and the results are shown in Fig. 3(C) and(D). The signal intensity of nitrogen(N) in the SCF-N treated needles was significantly higher than in the control group (2.3 ± 0.2% vs 0.0 ± 0.0%, *p* < 0.01). The higher proportion of nitrogen also resulted in a decreased proportion of (Fe), chromium (Cr), and nickel (Ni), which are the major constituents of the stainless steel needles.

### Modified visual analog scale score

The *de-qi* needle sensation was assessed using the modified visual analog scale (VAS) score as shown in Table 1. The four separate *de-qi* sensations and the cumulative *de-qi* sensation were analyzed, and the results showed that at LI4 and LI11, all volunteers experienced stronger *de-qi* sensations with the needles of the SCF-N group compared to those of the stainless steel needle control group. The sensation of soreness (2.76 ± 2.06 vs 2.13 ± 1.85, *P* = 0.045) and the combined *de-qi* sensation (1.87 ± 1.88 vs 1.54 ± 1.62, *P* = 0.014) at LI4 showed statistically significant differences. Study volunteers with SCF-N-treated needles tended to have stronger *de-qi* sensations, especially the sensation of soreness at LI4 acupoint, than with stainless steel needles.

**Table 1:** VAS score comparisons between SCF-N and Non-SCF-N group. Both LI4 and LI11 have stronger *de-qi* sensation on insertion of SCF-N treated needles as compared to the sensation with insertion of stainless steel needles. Soreness and the combined *de-qi* sensation of LI4 shows significant differences between the two groups.

Acupoint	Without SCF-N	With SCF-N	95% CI difference	*p* value
Hegu (LI4)				
*De-qi*	1.54 ± 1.62	1.87 ± 1.88	−0.07∼-0.59	0.014
Soreness	2.13 ± 1.85	2.76 ± 2.06	−0.02∼-1.25	0.045
Numbness	1.05 ± 1.13	1.47 ± 1.69	−0.10–0.92	0.110
Distension	1.59 ± 1.57	1.65 ± 1.71	−0.52–0.65	0.824
Heaviness	0.97 ± 1.33	1.26 ± 1.69	−0.18–0.76	0.223
Quchi (LI 11)				
*De-qi*	0.91 ± 1.13	1.06 ± 1.16	−0.35–0.05	0.142
Soreness	1.41 ± 1.44	1.67 ± 1.35	−0.24–0.76	0.308
Numbness	0.76 ± 0.89	0.87 ± 0.86	−0.21–0.41	0.506
Distension	0.75 ± 0.86	0.94 ± 1.04	−0.17–0.56	0.290
Heaviness	0.64 ± 0.89	0.82 ± 1.02	−0.19–0.55	0.325

Abbreviation: SCF-N: Supercritical fluid N modified.

### Electrical resistance

**Fig. 4:**
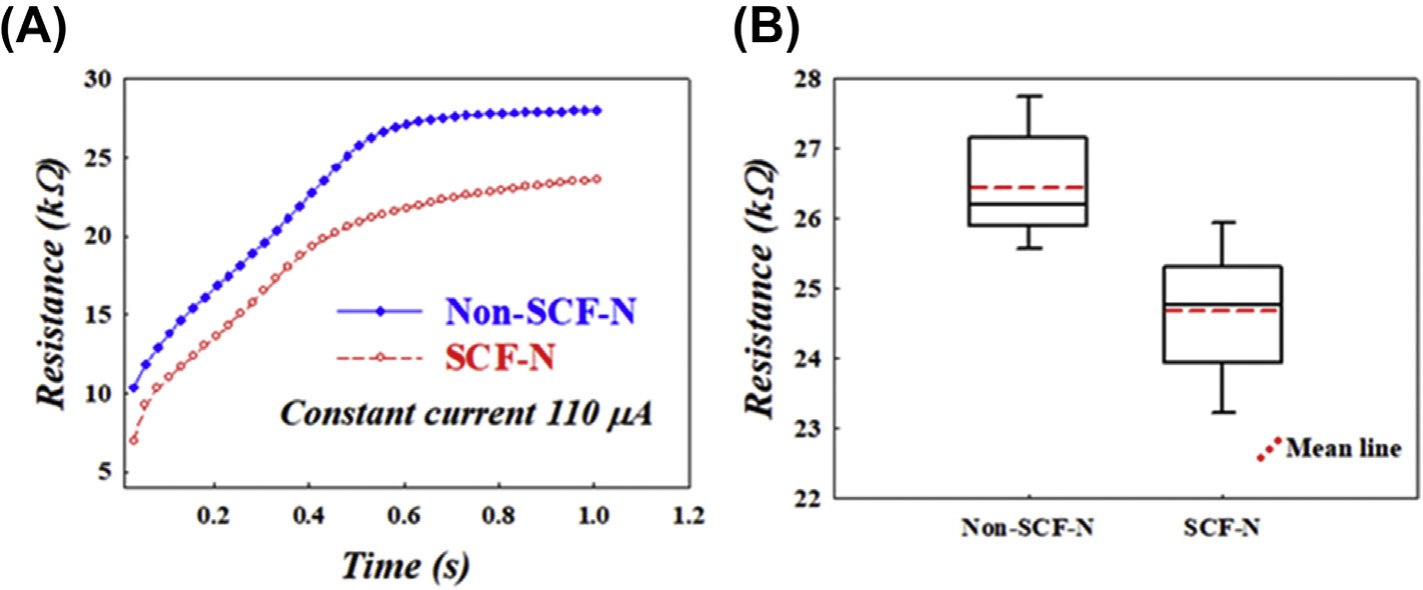
Comparison of electrical resistance in SCF-N treated and untreated control group. The electrical resistance 24.67 ± 0.88 kW in the SCF-N treated group is lower than 26.45 ± 0.75 kW for the control group.

In order to measure the electrical resistance between LI4 and LI11, the inserted needles were attached to an Agilent B1500 and an electrical current of 110 μA was passed through the needles. Fig. 4(A) shows that it took about 1 s to reach saturation and the electrical resistance of the SCF-N treated group was lower than that of the untreated group. According to Fig. 4(B), the average and median values of the electrical resistance of the SCF-N treated group showed the same trend across all 20 volunteers. According to Ohm's law, we can infer that the smaller the starting voltage, the smaller the contact resistance between the modified acupuncture needle and the human tissue. Our results indicated that SCF-N surface treatment of acupuncture needles can lower the electrical resistance between the human body and the needles (24.67 ± 0.88 kW vs 26.45 ± 0.75 kW, *P* < 0.01).

## Discussion

### Acupuncture needles modified with nitrogen-applied supercritical fluid

Through the good fluidity and solubility of supercritical fluid, acupuncture needles were modified with SCF-N in this study. The nitrogen-containing compound can be uniformly contacted with the acupuncture needle surface after being dissolved in the supercritical fluid. The main reason for choosing nitrogen rather than oxygen or carbon as the agent for surface treatment is that the use of nitrogen as an intermediate bridge makes it easier to combine two distinct substances with one another. It has better binding with functional groups than do carbon, oxygen, or other light elements. Moreover, NOx-will be produced at the surface of the needles during the SCF treatment process. SCF-N-treated needles helped reduce the resistance of meridian conduction in this study.

The SCF technology with gas-like diffusivity and liquid-like density in the supercritical phase has been used for extracting various metals and for surface modifications. It has been found to improve the solubility of metal chelates and chelating agents in medical materials [25,26]. Through enhancement of the manufacturing processes and bioavailability of materials, an SCF surface treatment may be an effective alternative for biomedical applications in a range of industrial and laboratory processes [[25], [26], [27]].

### Lowering the electrical resistance

We found that SCF-N treated acupuncture needles could improve *de-qi* sensations and decrease the meridian electrical resistance. There are many different meridian hypotheses, but they may roughly be classified as nerve conduction theory, body fluid circulation theory, fascia and connective tissue structure doctrine, and biological field (or energy) doctrine [28]. In the body fluid circulation theory, the material basis of the meridians are certain ions and neurotransmitters. Acupuncture can lead to ion conduction and transmission with drift and diffusion currents through the meridians [29]. Acupuncture and nitric oxide (NO) also plays an important role in this theory. According to a recent study, NO can be produced either enzymatically by NO synthases or non-enzymatically through nitrate-nitrite-NO pathways in our body [30,31]. Some research has found that NO has the ability to increase local blood flow [32,33]. Furthermore, it is also able to act as a neurotransmitter [34]. This has led to the development of 2- phenyl-4,4,5,5- tetra-methylimidazoline -1-oxyl 3-oxide (PTIO), a compound which can scavenge NO for use in biological systems [35,36], as well as the development of a painless, non-invasive biocapture device that uses PTIO to scavenge and quantify NO-related biomolecules over specific skin regions [30]. The concentrations of ${{\text{NO}}_{\text{x}}}^{-}$ and NO are physiologically released and generated from the skin surface of acupoints at high levels [37]. Acupuncture needle surface treated with nitrogen applied supercritical fluid may help enhance the signal of NO and the efficacy of acupuncture.

According to previous research, higher electrical resistance of the meridian is often accompanied by organ function or suboptimal health conditions [13]. Research has also shown that lower electrical resistance often represents a better connection to the meridian [13]. Our results showed that the electrical resistance of the SCF-N group was lower than that of the control group, which could indicate that using the SCF-N needles for electro-acupuncture may achieve a better therapeutic effect than using untreated needles. The EDS results showed an increased N signal with this increased N embedded as ${{\text{NO}}_{\text{x}}}^{-}$ on the surface of the SCF treated needles. Moreover, NO can act as a neurotransmitter, increasing the electrical conductivity of the meridian by decreasing the electrical resistance.

### Strengthening of *de-qi* sensation

*De-qi* is often associated with acupuncture needling sensations felt by the patient or perceived by the physician. *De-qi* sensations are associated with treatment effects of acupuncture in traditional Chinese medicine theory. Strong *de-qi* sensations, including distension, soreness, heaviness, or numbness, indicates a stronger acupuncture therapeutic effect. Everyone experiences different sensations after acupuncture; hence, we analyzed and compared the comprehensive sensation experienced between treatment with SCF-N-treated acupuncture needles and control stainless steel needles by the VAS score test. We found that both LI4 (Hegu acupoint) and LI11 (Quchi acupoint) revealed stronger *de-qi* sensation with SCF-N treated needles than with untreated stainless steel needles. Acupuncture with the SCF-N treated needles may have better therapeutic effect than with the control stainless steel needles. However, the difference was significant between the two groups only with regard to the soreness sensation and combined *de-qi* sensation at LI4. The sensation at LI11 showed no significant differences. The depth of the LI4 acupoint (0.5–0.8 cun) is shallower than that of the LI11 acupoint (1.0–1.5 cun). The biological and neural network around the LI4 acupoint is more abundant than that around the LI11 acupoint. Therefore, volunteers may experience *de-qi* sensation at LI4 acupoint more easily than at LI11 acupoint. Additionally, the results at LI11 may gain significance if the sample size were increased.

Acupuncture stimulation elicits a composite of the *de-qi* sensation. Some clinical acupuncture manipulations could induce the *de-qi* sensations, such as adjusting the correct acupuncture points; adjusting the direction and depth of acupuncture; massaging in the direction of the acupoint and meridian with hands; twisting, lifting, inserting, or shaking the acupuncture needle; using warm needling or moxibustion; and scraping the needle handle up and down. Occasionally, thicker needles are used to achieve the *de-qi* sensation. Based on our results, using the SCF-N-treated needles, we can achieve a more intense *de-qi* sensation without affecting the quality of the needle tip. In other words, thicker needles are no longer needed, reducing the incidence of bleeding and hematoma, which may in turn increase the patient's willingness to accept acupuncture therapy.

### Limitation

According to the theory of traditional Chinese medicine, twelve meridians act as the network in which qi and blood circulate throughout the body. LI4 and LI11 acupoints belong only to the Yangming Large Intestine Meridian of Hand. Further research is required to verify whether the other eleven meridians exhibit the same result.

## Conclusions

Acupuncture needle modified with SCF-N surface treatment can enhance *de-qi* sensations and improve electrical conductivity of the meridian and therapeutic effects on the Yangming Large Intestine Meridian of Hand. SCF-N-treated needles can be as a new acupuncture needle material. Future follow-up studies on other meridians will enable the more precise assessment of such SCF techniques applied to acupuncture needles.

## Compliance with ethics requirements

This clinical trial was approved by the Institutional Review Board of Chang Gung Medical Foundation (IRB permit no. 201700722A3).

## Conflicts of interest

The authors have declared no conflict of interest.
